# Tracking and Estimation of Multiple Cross-Over Targets in Clutter

**DOI:** 10.3390/s19030741

**Published:** 2019-02-12

**Authors:** Sufyan Ali Memon, Myungun Kim, Hungsun Son

**Affiliations:** 1 Department of Electrical Engineering, Indus University, Karachi 75300, Pakistan; sufyanahmedali@gmail.com; 2 Mechanical, Aerospace and Nulcear Engineering, Ulsan National Institute of Science & Technology, Ulsan 44919, Korea; mgkim3070@unist.ac.kr

**Keywords:** cross-over targets, estimation, false-track discrimination (FTD), smoothing, tracking

## Abstract

Tracking problems, including unknown number of targets, target trajectories behaviour and uncertain motion of targets in the surveillance region, are challenging issues. It is also difficult to estimate cross-over targets in heavy clutter density environment. In addition, tracking algorithms including smoothers which use measurements from upcoming scans to estimate the targets are often unsuccessful in tracking due to low detection probabilities. For efficient and better tracking performance, the smoother must rely on backward tracking to fetch measurement from future scans to estimate forward track in the current time. This novel idea is utilized in the joint integrated track splitting (JITS) filter to develop a new fixed-interval smoothing JITS (FIsJITS) algorithm for tracking multiple cross-over targets. The FIsJITS initializes tracks employing JITS in two-way directions: Forward-time moving JITS (fJITS) and backward-time moving JITS (bJITS). The fJITS acquires the bJITS predictions when they arrive from future scans to the current scan for smoothing. As a result, the smoothing multi-target data association probabilities are obtained for computing the fJITS and smoothing output estimates. This significantly improves estimation accuracy for multiple cross-over targets in heavy clutter. To verify this, numerical assessments of the FIsJITS are tested and compared with existing algorithms using simulations.

## 1. Introduction

In noisy surveillance situation, multiple cross-over targets tracking (MTT) is a difficult task facing various complexities [1]. Sensors such as radar returns measurements to the tracking system which may be spurious (clutter) due to the presence of various object’s sources for example, target thermal noise, clouds and reflections from terrain and so forth. Such clutter measurements result in inaccurate estimates [1]. Moreover, the target tracker cannot determine the target motions without any prior information. In addition, it results in the uncertain target motion with a low detection probability *P_d_*. Thus, MTT is a challenging issue due to the lack of true and fast track management. Therefore, an effective track management technique is required for practical applications such as military, threat assessment and awareness systems [2].

The MTT algorithms use an automatic track initialization procedure which results in both false (clutter) and true tracks (target). For an automatic track maintenance, MTT employed false track discrimination (FTD) [2] technique which uses track quality measure to identify and confirm, true and false tracks. There are two ways for MTT and estimation: (1) non-smoothing and (2) smoothing. The non-smoothing algorithms estimate the target state in a current scan only with current scan measurement based on a number of methods such as Monte-Carlo data association [3], joint probabilistic data association (JPDA) [4,5,6,7,8], probability hypothesis density [9], multi-hypothesis tracking [10,11], joint integrated PDA (JIPDA) [12,13] and joint integrated track splitting (JITS) [14,15]. However, only JIPDA and JITS provide track quality measurement for the FTD using probability of target existence. The ITS [16] splits a track into multiple probability density function (pdf) components (accompanied with mean and covariance of measurement) and employs the component measurement histories for association to the track. Hence, ITS is computationally more accurate for estimation than IPDA [17,18]. The term “joint” used in JIPDA/JITS indicates that measurements are shared between tracks or similar measurements are assigned to tracks which form the cluster measurements. The joint measurement has no sign of target detection, however if this measurement is a target detection, it may changes the possibility of the measurement outcome from other objects (target/clutter). Thus, MTT or joint data association algorithm generates growing number of possible hypotheses (which termed as possible joint event (PJE)) which must be allocated to possible tracks jointly or globally. PJE is an event which enumerates joint measurement-to-track assignments and evaluates their a-posteriori probabilities. In principle, the number of PJEs grows combinatorically with the number of tracks and the number of target measurements. Therefore, MTT utilizes the clustering control approach described in References [2,3] which groups the possible tracks in a cluster to limit the computational complexities. Unlike to single target tracker, the joint multi-target data association probabilities are obtained by using a-posteriori probabilities of the measurements and target detection probabilities.

Smoothing utilizes the measurement from future scans to obtain the estimate in current scan which results in the reduction of estimation errors [2] and [19]. The smoothing algorithms include fixed-lag smoothing based on JPDA [20], Gaussian sum smoothing [21], fixed-interval smoothing JIPDA (JIPDAS) [22] and smoothing JITS (sJITS) [23] where only JIPDAS and sJITS provide FTD with smoothing probability of target existence. In (22), a fixed-lag smoothing data association based on IPDA [24] algorithm is extended in MTT environment using JIPDA. Another version of ITS family is known as fixed-interval ITS smoothing (ITS-S) [25,26] but it is developed only for single target tracking (STT).

**Figure 1:**
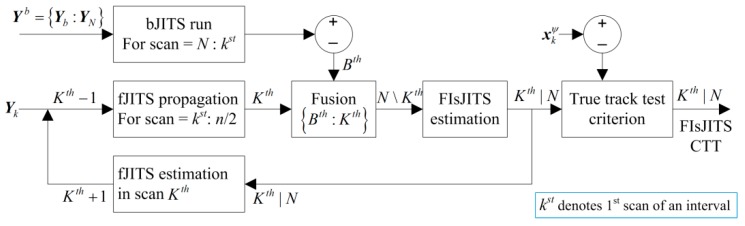
Block Diagram of Feedback-loop tracking for one interval.

sJITS [23] utilize the validation measurements and their joint data association probabilities obtained from forward JITS tracking (fJITS) for backward JITS (bJITS) track estimation. Thus in sJITS, each backward track corresponds to each forward track for fusion. However, this paper proposes the feedback-loop tracking to develop a new smoothing algorithm called the fixed-interval smoothing JITS (FIsJITS). For brief description of the proposed algorithm, the fixed-interval smoothing is illustrated for one interval only as shown in Figure 1. This novel feedback-loop technique fetches the measurements from future scans using bJITS multi-tracks estimations to compute FIsJITS and fJITS estimates in the past *K*th scan. The fixed-interval has a length of n and has N − k + 1 scans. The bJITS block depicted in Figure 1 utilizes JITS in backward-time direction where measurements ***Y**^b^* are applied for backward track initialization. The bJITS iterates in a smoothing interval starting from scan N ending to the *B*th scan for backward multi-track component recursive estimations using ***Y**_b_* in each scan. When the bJITS tracks arrived in the *K*th scan (*B*th = *K*th), fJITS propagation block in Figure 1 initialize forward tracks using measurements ***Y**_k_* to obtain forward components and propagate them for fusion. In the fusion block, each of the fJITS component prediction creates a validation gate assuming bJITS multi-track predictions as measurements in the forward-path. The junction block connected to the fusion block indicates the backward measurement selection [24] in the forward-path track which subtracts the backward and forward predictions and validates bJITS components for fusion. This fusion generates multiple validated smoothing component predictions associated to validated bJITS components and thus, a predicted FIsJITS track component is formed for smoothing in the *N*\*K*th scan. Scan index *N*\*K*th indicates that the smoothing component prediction is calculated based on backward measurements and that is not conditioned on ***Y**_k_*. The smoothing component predictions *N*\*K*th are applied to FIsJITS block to obtain smoothing component estimates in the *K*th scan conditioning on ***Y**_N_* as indicated by *K*th|*N* in Figure 1. The clusters and PJEs are formed in FIsJITS track to evaluate smoothing target existence, joint data association and component existence probabilities. This approximates the FIsJITS track by one Gaussian pdf smoothing mean and covariance. The FIsJITS state estimate mean must be validated through target state in the *K*th scan using the true track test criterion [17] to obtain a FIsJITS confirmed true track (CTT) output. For computational efficiency, a feedback-loop is created where the smoothing joint data association probabilities *K*th|*N* are utilized in fJITS for forward-time multi-tracks estimation in the *K*th scan. Consequently, each existing forward track component estimate is propagated to the next scan in fJITS propagation block and concatenated to the newly initialized forward tracks required for fusion in scan *K*th + 1 as depicted in Figure 1. This efficiently makes the forward track robust for target tracking. Similarly, when the FIsJITS track arrived at half of an interval n/2 scan, the next interval needs to overlap in the current interval to develop the subsequent overlapped measurement-intervals for smoothing. Therefore, both fJITS and FIsJITS run from scan *K*th to scan index at n/2 in each subsequent interval except the last interval, where they run for all scans. The numerical assessment is verified with simulations to reinforce the FIsJITS algorithm. 

## 2. Target Model

It is assumed that a target tracking sensor could provide a measurement from each target per each scan. Nevertheless, it could lose the target or track a wrong target (or clutter) under heavy clutter environment, since the tracker does not know any priory information on the measurements. Therefore, the originated measurement may relate to a target or clutter. Denote the target label as well as track label by *ψ*. Without loss of generality, suppose that tracker measure velocity and position in the 2-dimensional (2D) surveillance region with an infinite resolution sensor. The 4D state vector (composed of 2D position and velocity vectors) of the target $x_{k}^{\psi }$ can be estimated from the predicted state vector. The state prediction is obtained by propagating $x_{k}^{\psi }$ from scan k−1 to scan k in Equation (1).

(1) $$x_{k}^{\psi }=F_{k-1}x_{k-1}^{\psi }+v_{k-1}$$

where the kinematic model $F_{k-1}$ in (2) is employed for propagation and $v_{k-1}$ is kinematic model uncertainty with a zero-mean and a covariance $Q_{k-1}$ of the white Gaussian in Equation (3).

(2) $$F_{k-1}=\left[\begin{array}{cc} I_{2\times 2} & TI_{2\times 2}\\ O_{2\times 2} & I_{2\times 2} \end{array}\right]$$

and

(3) $$Q_{k-1}=\upsilon \left[\begin{array}{cc} T^{4}I_{2\times 2}/3 & T^{3}I_{2\times 2}/2\\ T^{3}I_{2\times 2}/2 & T^{2}I_{2\times 2} \end{array}\right]$$

where υ represents a variance constant due to target motion uncertainty [24] and *T* represents the time scan. Track has a hybrid state composed of target state and target existence event $\chi_{k}^{\psi }$. The *ψ*th target position measurement $z_{k}^{\psi }$ in the *K*th scan is expressed in Equation (4). In tracking gate, if target exist and detected with detection probability *P_d_*, label the existing track by *ψ*th in each scan.

(4) $$z_{k}^{\psi }=H_{k}x_{k}^{\psi }+w_{k}$$

where ***H**_k_* collects at-most one position measurement from the *ψ*th target for data association in each scan and *w_k_* is the white Gaussian target measurement noise with a zero-mean and a known covariance matrix ***R**_k_* received in the *K*th scan.

## 3. Smoothing Multi-Target Using Joint Integrated Track Splitting (FIsJITS)

The FIsJITS algorithm is the extension of ITS-S [25] in MTT environment which employs JITS with the smoothing data association for tracking multiple cross-over targets in heavy clutter. 

### 3.1. Backward Joint Integrated Track Splitting (bJITS)

FIsJITS utilizes JITS in the reverse direction of an interval from the *N*th scan to the *B*th scan to develop multi-track bJITS estimation. The bJITS tracks are formed and updated in each scan of an interval ***Y^b^*** = [***Y****_b_*, ***Y***_*b*+1_, …, ***Y****_N_*_−1_, ***Y****_N_*]. The two-point track initialization [17] procedure is applied which uses each pair of measurement taken from two consecutive scans to initialize a new backward track. For example; a pair of measurement from ***Y**_N_* and ***Y**_N_*_−1_ initializes a track which provides the updated track component pdf $p\left\{\chi_{b+1}^{\psi },X_{b+1}^{\psi }|Y_{b+1}\right\}$ based on ***Y**_b_*_+1_. Each new track has an initialized component existence probability $\zeta_{b+1}^{\psi }=1$ and an initial target existence probability. The component pdf propagates using Equation (5).

(5) $$\left[{\bar{X}}_{b+1}^{\psi },{\bar{P}}_{b+1}^{\psi }\right]={\mathrm{KF}}_{\mathrm{Pre}}\left({\hat{X}}_{b+1}^{\psi },{\hat{P}}_{b+1}^{\psi },F_{b+1},Q_{b+1}\right)$$

where **KF**_Pre_ denotes the Kalman filter prediction [24], hat accent (^) indicates state estimate and bar accent (−) indicates state prediction. $F_{b+1}=F_{k-1}^{-1}$ and $Q_{b+1}=F_{k-1}^{-1}Q_{k-1}F_{k-1}^{-T}$ (where superscript −T denotes inverse transpose) are used for state propagation. 

The bJITS multi-track component prediction in Equation (5) is applied to the measurement selection criterion [17] expressed in Equation (6) in order to select the validation component measurement ***y****_b_*_,*i*_ from ***Y****_b_* in the *B*th scan. This generates a validation gate around a component prediction and some possible clutter measurements which satisfy Equation (6).

(6) $$\sigma_{b,i}^{T}S^{-1}\sigma_{b,i}\leq \eta$$

where η is the gating limit determined from the gating probability [17] (expressed by *P_g_* = 1 − e^−0.5*η*^) and ***σ**_b_*_,*i*_ and ***S*** express the innovation and covariance of the measurement ***Y****_b_*_,*i*_ in Equations (7) and (8) respectively.

(7) $$\sigma_{b,i}=Y_{b,i}-H_{k}{\bar{X}}_{b+1}^{\psi }$$

and

(8) $$S=H_{k}{\bar{P}}_{b+1}^{\psi }H_{k}^{T}+R_{k}$$

The component prediction calculates component likelihood measurement $l_{b,i}^{\psi }$ of ***y****_b_*_,*i*_ in Equation (9).

(9) $$l_{b,i}^{\psi }=N\left(y_{b,i};H_{k}{\bar{X}}_{b+1}^{\psi },S\right)/P_{g}$$

**Figure 2:**
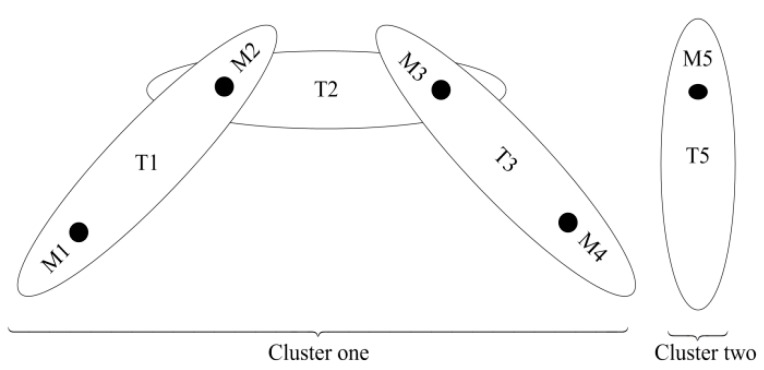
Formation of clusters.

To avoid computational complexity, separate the tracks in the form of clusters. The clusters are formed: (1) when the track shares measurement ***y****_b_*_,*i*_ with neighboured tracks and (2) when cluster track does not share its measurement ***y****_b_*_,*i*_ with any other neighboured tracks. Each cluster processes independently and simultaneously. Figure 2 shows formation of two clusters in the *B*th scan where ellipsoids are indicating validation gates for respective cluster tracks. In cluster one; there are three tracks from T1 to T3 selecting four validation measurements from M1 to M4 respectively. In this joint measurement situation, measurement M2 is assigned and shared to both tracks T1 and T2, in addition, measurement M3 is shared to both tracks T2 and T3. However, the measurements M1 and M4 assigned to tracks T1 and T3 respectively are possibly resulted from clutter. Track T5 does not share its measurement M5 with any other neighboured track forms a separate cluster. The allocation of such validated measurements to tracks is enumerated using Equation (10) for the evaluation of their a-posteriori probabilities using Equation (11) in (10). Each measurement allocation expresses a possible joint event (PJE) represented by *p*(*ε_i_*|***Y****_b_*) in Equation (10). A PJE is described in Reference [14] as one possible mapping of all validation measurements to all tracks in a cluster such that it assigns at-most one validated measurement to each cluster track. The set of PJEs is exclusive and exhaustive; therefore, only one PJE is correct.

(10) $$p(\varepsilon_{i}|Y_{b})=G^{-1}\prod_{\psi \in t_{o}^{i}(\varepsilon_{i})}\left(1-\mu \right)\times \prod_{\psi \in t_{1}^{i}(\varepsilon_{i})}\left(\mu \frac{l_{b}^{\psi }}{\rho_{b,i}}\right)$$

which allocates set of tracks with $t_{0}^{i}$ and $t_{1}^{i}$ for assigning measurements for *i* = 0 and *i* > 0, respectively and calculates their a-posteriori probabilities using Equation (11) in (10). *ρ_b_*_,*i*_ in Equation (10) denotes the clutter measurement density in the *B*th scan.

(11) $$\mu =P_{d}P_{g}\alpha {\bar{\delta }}_{b+1}^{\psi }$$

where α denotes the target state transition probability which updates the predicted bJITS target existence probability ${\bar{\delta }}_{b+1}^{\psi }$. The bJITS track likelihood measurement $l_{b}^{\psi }$ in Equation (10) is the Gaussian summed-up of component likelihood measurements expressed in Equation (12).

(12) $$l_{b}^{\psi }=p\left(y_{b,i}|Y_{b+1}\right)=\sum_{i}\zeta_{b+1}^{\psi }l_{b,i}^{\psi }$$

and *G* in Equation (10) is normalization constant which must ensures that

(13) $$\sum_{\varepsilon_{i}}P\left\{\varepsilon_{i}|Y_{b}\right\}=1$$

The PJE calculates the a-posteriori probability of the no validated measurement ***y****_b_*_,*i*_ originating from target is expressed in Equation (14). This indicates that Equation (14) is a-posteriori probability of the clutter measurement assigned to a cluster track.

(14) $$P\left\{\chi_{b,0}^{\psi }|Y_{b}\right\}=\sum_{\varepsilon \in (\psi ,i=0)}P\left\{\varepsilon_{i}|Y_{b}\right\}$$

Equation (14) is used to compute the bJITS *ψ*th target non-existence probability in Equation (15).

(15) $$P\left\{\chi_{b}^{\psi },\chi_{b,0}^{\psi }|Y_{b}\right\}=\frac{\left(1-P_{d}P_{g}\right)\alpha {\bar{\delta }}_{b+1}^{\psi }}{1-\mu }P\left\{\chi_{b,0}^{\psi }|Y_{b}\right\}$$

Simultaneously, the PJE calculates the a-posteriori probability of the validated measurement ***y****_b_*_,*i*_ originating from target using Equation (10) in Equation (16). This indicates that the shared measurements M2 (shared to T1 and T2) and M3 (shared to T2 and T3) depicted in Figure 2, have the a-posteriori probability equal to Equation (16). Table 1 describes the measurements allocation to tracks and their a-posteriori probabilities.

(16) $$P\left\{\chi_{b}^{\psi },\chi_{b,i}^{\psi }|Y_{b}\right\}=\sum_{\varepsilon \in (\psi ,i>0)}P\left\{\varepsilon_{i}|Y_{b}\right\}$$

Table 1 lists 21 PJEs as described in Reference [2]. In first PJE, there is no measurement assigned to tracks and thus all measurements are declared as clutter. Therefore, a-posteriori probabilities of null (zero) measurements (i.e., the measurement not originated from target) in the event *p*(*ε*_1_|***Y****_b_*) are calculated using (14). In *p*(*ε*_2_|***Y****_b_*), measurement M1 and in *p*(*ε*_2_|***Y****_b_*), measurement M2 is assigned to track T1, whereas a null measurement is assigned to tracks T2 and T3. Thus, Equation (16) is used to calculate the a-posteriori probability of M1 and M2, enumerated in *p*(*ε*_1_|***Y****_b_*) and *p*(*ε*_2_|***Y****_b_*), respectively. While, Equation (14) calculates the a-posteriori probability of no measurement selected from targets allocated to T2 and T3 in *p*(*ε*_2_|***Y****_b_*) and *p*(*ε*_2_|***Y****_b_*), respectively. PJE *p*(*ε*_21_|***Y****_b_*) assigns the measurements M2, M3 and M4 to track T1, T2 and T3, respectively and M1 is declared as clutter measurement. Thus, the sum of a-posteriori probabilities of all measurements (*i* ≥ 0) is the bJITS estimated probability of the *ψ*th target existence in *B*th scan expressed in Equation (17).

(17) $${\hat{\delta }}_{b}^{\psi }=\sum_{\varepsilon \in (\psi ,i\geq 0)}P\left\{\chi_{b}^{\psi },\chi_{b,i}^{\psi }|Y_{b+1}\right\}$$

Consequently, the bJITS multi-target component data association probabilities of PJEs associated to the track is computed using Equation (17) in Equation (18).

(18) $$\beta_{b}^{\psi }=\frac{P\left\{\chi_{b}^{\psi },\chi_{b,i}^{\psi }|Y_{b+1}\right\}}{{\hat{\delta }}_{b}^{\psi }}$$

which creates a new track component with a PJE measurement *i* ≥ 0 and calculates its’ probability of component existence in Equation (19).

(19) $$\zeta_{b}^{\psi }=\beta_{b}^{\psi }\zeta_{b+1}^{\psi }\{\begin{array}{l} 1;\;i=0\\ \frac{l_{b,i}^{\psi }}{l_{b}^{\psi }};\;i>0 \end{array}$$

The predicted backward track component obtained from Equation (5) is estimated using Kalman filter (KF) in Equation (20) based on the *B*th scan validated measurement ***y****_b_*_,*i*_.

(20) $$\left[{\hat{X}}_{b}^{\psi },{\hat{P}}_{b}^{\psi }\right]={\mathrm{KF}}_{\mathrm{Est}}\left(y_{b,i},R_{k},{\bar{X}}_{b+1}^{\psi },{\bar{P}}_{b+1}^{\psi }\right)$$

where subscript on KF indicates estimation. In the next *B*th scan, the backward state prediction (state retrodiction) would be the result of Equation (20) which could be retrieved from scan *b* + 1 using Equation (5). Similarly, the procedure iterates from Equation (5) to Equation (20) for recursive bJITS multi-track estimation in each scan.

### 3.2. Forward Joint Integrated Track Splitting (fJITS)

In scan *B*th ≥ *K*th, the two-point track formation [17] is again used such that each pair of measurement in ***Y****^k^* initializes a new forward track which computes the component state pdf $p\left\{\chi_{k-1}^{\psi },X_{k-1}^{\psi }|Y_{k-1}\right\}$ conditioned on ***Y****_k_*_−1_. The fJITS track component pdf is propagated using **KF**_Pre_ to obtain the fJITS component prediction conditioned on ***Y****_k_*_−1_ in Equation (21). Each track has an initial fJITS target existence probability ${\bar{\delta }}_{k-1}^{\psi }$ and has an initial fJITS component existence probability $\zeta_{k-1}^{\psi }=1$.

(21) $$\left[{\bar{X}}_{k-1}^{\psi },{\bar{P}}_{k-1}^{\psi }\right]={\mathrm{KF}}_{\mathrm{Pre}}\left({\hat{X}}_{k-1}^{\psi },{\hat{P}}_{k-1}^{\psi },F_{k-1},Q_{k-1}\right)$$

FIsJITS utilizes the assumption of ITS-S without using unnecessary data association in the fusion. Exploiting bJITS multi-track component predictions as a set of measurements in a forward track validates the bJITS component for fusion using the validation gate selection criterion expressed in Equation (22). Further assume that each backward track and backward component is a mutually exclusive measurement.

(22) $${\left({\bar{X}}_{b+1}^{\psi }-{\bar{X}}_{k-1}^{\psi }\right)}^{T}{\left({\bar{P}}_{b+1}^{\psi }+{\bar{P}}_{k-1}^{\psi }\right)}^{-1}\left({\bar{X}}_{b+1}^{\psi }-{\bar{X}}_{k-1}^{\psi }\right)\leq \eta$$

**Figure 3:**
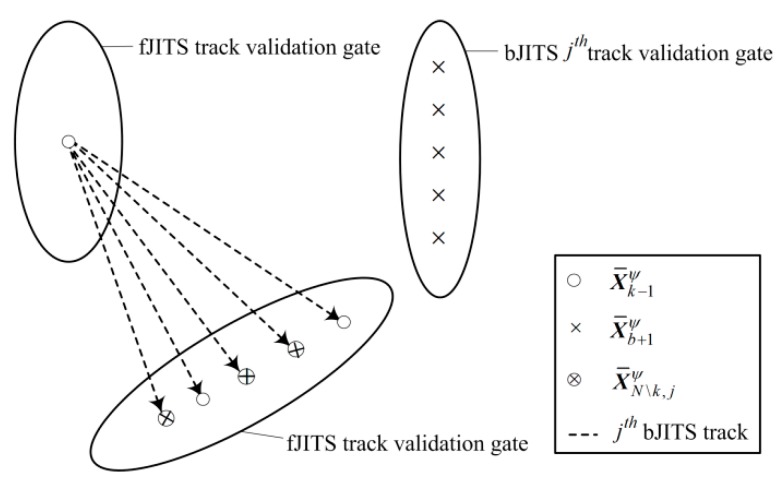
Fusion of a fJITS prediction with bJITS predictions.

fJITS track forms a validation gate using Equation (22) and selects bJITS validation component prediction for fusion to obtain the predicted smoothing component ${\bar{X}}_{N\backslash k,j}^{\psi }$ using an information fusion (IF) filter [25] in Equation (23a). However, if the backward track (labelled by *j*th) is not validated, the smoothing component prediction becomes a forward component as expressed in Equation (23b). Figure 3 illustrates this fusion where “circle” indicates a forward component, “cross” indicates backward component and “cross-in-circle” indicates smoothing component prediction.

(23a) $$\left[{\bar{X}}_{N\backslash k,j}^{\psi },{\bar{P}}_{N\backslash k,j}^{\psi }\right]=\mathrm{IF}\left({\bar{X}}_{b+1}^{\psi },{\bar{X}}_{k-1}^{\psi },{\bar{P}}_{b+1}^{\psi },{\bar{P}}_{k-1}^{\psi }\right)$$

(23b) $$\left[{\bar{X}}_{N\backslash k,j}^{\psi },{\bar{P}}_{N\backslash k,j}^{\psi }\right]=\left[{\bar{X}}_{k-1}^{\psi },{\bar{P}}_{k-1}^{\psi }\right]$$

Each fJITS track computes the hybrid component measurement likelihood associated to *j*th validated bJITS track component in Equation (24). Otherwise, $l_{k,(b,j)}^{\psi }=0$.

(24) $$l_{k,(b,j)}^{\psi }=\frac{1}{P_{g}}N\left({\bar{X}}_{b+1}^{\psi },{\bar{X}}_{k-1}^{\psi },{\bar{P}}_{b+1}^{\psi }+{\bar{P}}_{k-1}^{\psi }\right)$$

which is required to calculate the predicted smoothing component existence probability in Equation (25a) for the *j*th validated track. However, if the *j*th track is not validated, then the predicted smoothing component existence probability becomes Equation (25b).

(25a) $$\zeta_{N\backslash k}^{\psi }=\frac{\partial \zeta_{k-1}^{\psi }\zeta_{b+1}^{\psi }}{\lambda_{N\backslash k}}\left(\frac{l_{k,(b,j)}^{\psi }}{d_{b,j}}\right)$$

(25b) $$\zeta_{N\backslash k}^{\psi }=\frac{\left(1-\partial \right)\zeta_{k-1}^{\psi }}{\lambda_{N\backslash k}}$$

where a new factor ∂ = *P_g_* − *P_g_*(1 − *P_d_*)^*N*−*k* + 1^ defines that the *ψ*th target existence is detected in an interval, *d_b_*_,*j*_ = *f_b_*/**A** (where *f_b_* indicates the number of backward false-tracks and **A** denotes the surveillance area) represents the density of the initialized bJITS multi-tracks, *λ_N_*_\*k*_ is the modified hybrid track likelihood ratio expressed in Equation (26).

(26) $$\lambda_{N\backslash k}=1-\partial +\partial \sum_{j}\frac{l_{k,(b,j)}^{\psi }}{d_{b,j}}{\hat{\delta }}_{b}^{\psi }$$

where ${\hat{\delta }}_{b}^{\psi }$ and *∂* are used to update predicted FIsJITS track since the possible *ψ*th target state is already estimated in Equation (20) at the *B*th scan. This modification in the hybrid track likelihood ratio (which is not addressed in ITS-S) makes an effective way to calculate the predicted smoothing component existence probability in Equation (25) and the predicted smoothing target existence probability in Equation (27) at the *N*\*K*th scan.

(27) $${\bar{\delta }}_{N\backslash k}^{\psi }=\frac{\lambda_{N\backslash k}\alpha {\bar{\delta }}_{k-1}^{\psi }}{1-\left(1-\lambda_{N\backslash k}\right)\alpha {\bar{\delta }}_{k-1}^{\psi }}$$

### 3.3. Fixed-Interval Smoothing JITS (FIsJITS) and fJITS Estimations

FIsJITS computes both the smoothing estimates and forward estimates. Both computations require the smoothing validation measurements ${\tilde{y}}_{k,i}\in Y_{k,i}$ selected by using the predicted smoothing component obtained from Equation (23) in Equation (28).

(28) $${\left(Y_{k,i}-H_{k}{\bar{X}}_{N\backslash k,j}^{\psi }\right)}^{T}S^{-1}\left(Y_{k,i}-H_{k}{\bar{X}}_{N\backslash k,j}^{\psi }\right)\leq \eta$$

where $S=H_{k}{\bar{P}}_{N\backslash k,j}^{\psi }H_{k}^{T}+R_{k}$. The smoothing component prediction is used to compute the smoothing component measurement likelihood and smoothing track likelihood measurement based on ${\tilde{y}}_{k,i}$ in Equation (29) and Equation (30) respectively.

(29) $$l_{N\backslash k,i}^{\psi }=N\left({\tilde{y}}_{k,i};H_{k}{\bar{X}}_{N\backslash k,j}^{\psi },S^{-1}\right)/P_{g}$$

and

(30) $$l_{N\backslash k}^{\psi }=p\left({\tilde{y}}_{k,i}|Y_{N\backslash k}\right)=\sum_{i}\zeta_{N\backslash k}^{\psi }l_{N\backslash k,i}^{\psi }$$

Like bJITS, FIsJITS separates the tracks in the form of clusters and maps all possible validated smoothing measurement-to-track assignments for multi-target data association evaluation in the cluster. The smoothing PJE conditioned on ***Y****_N_*, *p*(*ε_i_*|***Y****_N_*) assigns the measurement ${\tilde{y}}_{k,i}\in z_{k}^{\psi }$ and ${\tilde{y}}_{k,i}\notin z_{k}^{\psi }$ to FIsJITS track using Equation (30) replacing $l_{b}^{\psi }$ and $\mu =P_{d}P_{g}{\bar{\delta }}_{N\backslash k}^{\psi }$ in Equation (10) to calculate the a-posteriori probability of the measurement in the event *ε_i_*. Similarly, all cluster PJEs are enumerated for $t_{0}^{i}$ (*i* = 0) and $t_{1}^{i}$ (*i* > 0) respectively, in Equation (10) to calculate the *ψ*th smoothing target existence probability and multi-target data association probability in Equations (31) and (32) respectively.

(31) $${\hat{\delta }}_{k|N}^{\psi }=\sum_{i\geq 0}P\left\{\chi_{k}^{\psi },\chi_{k,i}^{\psi }|Y_{N}\right\}$$

and

(32) $$\beta_{k|N}^{\psi }=\frac{P\left\{\chi_{k}^{\psi },\chi_{k,i}^{\psi }|Y_{N}\right\}}{{\hat{\delta }}_{k|N}^{\psi }}$$

where subscript *N* on ***Y****_N_* indicates that a target existence event is now conditioned on ***Y****_N_*. Equation (32) implies that the data (measurement) association probability is proportional to the a-posteriori probability of the measurement ${\tilde{y}}_{k,i}$ which is an important metric for calculating the smoothing and forward estimates in the *K*th scan. Note that smoothing components do not propagates, therefore Equation (10) does not uses α in *μ* for calculating a-posteriori probability of the measurement ${\tilde{y}}_{k,i}$.

Each smoothing component and a PJE measurement form a new smoothing component with smoothing component existence probability calculated using Equation (32) in Equation (33).

(33) $$\zeta_{k|N}^{\psi }=\beta_{k|N}^{\psi }\zeta_{N\backslash k}^{\psi }\{\begin{array}{l} 1;\;i=0\\ \frac{l_{N\backslash k,i}^{\psi }}{l_{N\backslash k}^{\psi }};\;i>0 \end{array}$$

FIsJITS computes the smoothing track component state estimate based on validated smoothing measurements ${\tilde{y}}_{k,i}$ using KF estimator in Equation (34).

(34) $$\left[{\hat{X}}_{k|N,j}^{\psi },{\hat{P}}_{k|N,j}^{\psi }\right]={\mathrm{KF}}_{\mathrm{Est}}\left({\tilde{y}}_{k,i},R_{k},{\bar{X}}_{N\backslash k,j}^{\psi },{\bar{P}}_{N\backslash k,j}^{\psi }\right)$$

Equation (33) approximate the smoothing track components by one Gaussian pdf smoothing mean and covariance in Equations (35) and (36) respectively.

(35) $${\hat{X}}_{k|N}^{\psi }=\sum_{k|N}\zeta_{k|N}^{\psi }{\hat{X}}_{k|N,j}^{\psi }$$

(36) $${\hat{P}}_{k|N}^{\psi }=\sum_{k|N}\zeta_{k|N}^{\psi }\left({\hat{P}}_{k|N,j}^{\psi }+{\hat{X}}_{k|N,j}^{\psi }{\hat{X}}_{k|N,j}^{\psi }{}^{T}\right)-{\hat{X}}_{k|N}^{\psi }{\hat{X}}_{k|N}^{\psi }{}^{T}$$

Unlike the existing algorithms (e.g., JIPDAS and sJITS), PJE’s assignments are not necessary in the forward path and the clusters formed by FIsJITS track are used for assigning smoothing measurements to the fJITS track. In other words, the smoothing measurements (${\tilde{y}}_{k,i}\in z_{k}^{\psi }$ and/or ${\tilde{y}}_{k,i}\notin z_{k}^{\psi }$) selected from Equation (28) are used to estimate fJITS components. Therefore, the likelihood of fJITS track component prediction conditioned on ***Y****_k_*_−1_ is calculated by using ${\tilde{y}}_{k,i}$ in Equation (37).

(37) $$l_{k,i}^{\psi }=N\left({\tilde{y}}_{k,i};H_{k}{\bar{X}}_{k-1}^{\psi },H_{k}{\bar{P}}_{k-1}^{\psi }H_{k}^{T}+R_{k}\right)/P_{g}$$

To solve multi-target data association in fJITS, the smoothing component data association probability $\beta_{k|N}^{\psi }$ is utilized as a weight for the likelihood of each forward track in Equation (38).

(38) $$l_{k}^{\psi }\equiv p\left({\tilde{y}}_{k,i},{\bar{X}}_{k-1}^{\psi }|Y_{k-1}\right)=\beta_{k|N}^{\psi }\sum_{i}\zeta_{k-1}^{\psi }l_{k,i}^{\psi }$$

The fJITS component existence probability $\zeta_{k}^{\psi }$ in the *K*th scan is calculated by Equations (37) and (38) and $\zeta_{k-1}^{\psi }$ in Equation (33) replacing $l_{k|N,i}^{\psi }$, $l_{k|N}^{\psi }$ and $\zeta_{N\backslash k}^{\psi }$ respectively. This reinforces the forward track for tracking targets efficiently and the results are verified in Section 4. The fJITS *ψ*th target existence probability ${\hat{\delta }}_{k}^{\psi }$ is updated in Equation (39) using modified forward track likelihood ratio expressed in Equation (40).

(39) $${\hat{\delta }}_{k}^{\psi }=\frac{\lambda_{k}\alpha {\bar{\delta }}_{k-1}^{\psi }}{1-\left(1-\lambda_{k}\right)\alpha {\bar{\delta }}_{k-1}^{\psi }}$$

(40) $$\lambda_{k}=1-\partial +\partial \sum_{i}\frac{l_{k}^{\psi }}{\rho_{k,i}}$$

**Figure 4:**
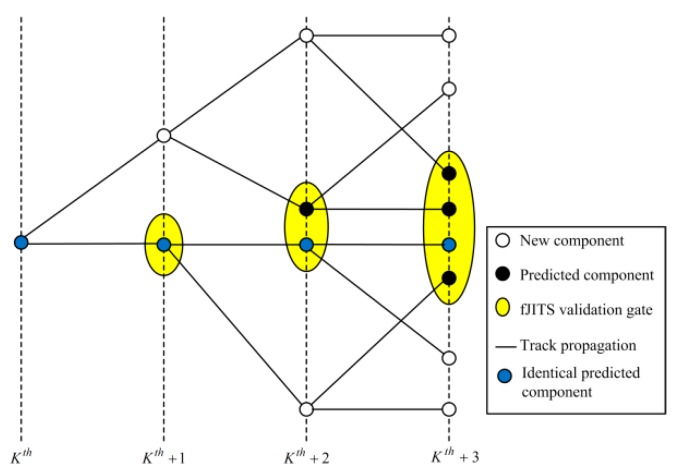
Component propagations and merging in forward-path track.

Similarly, the predicted fJITS component mean and covariance obtained from Equation (21) are used based on ${\tilde{y}}_{k,i}$ by replacing ${\bar{X}}_{N\backslash k,j}^{\psi }$ and ${\bar{P}}_{N\backslash k,j}^{\psi }$ in Equation (34) to obtain the fJITS track component estimate ${\hat{X}}_{k}^{\psi }$ and ${\hat{P}}_{k}^{\psi }$. $\zeta_{k}^{\psi }$ and $\zeta_{b}^{\psi }$ are used to approximate the forward and backward tracks by one Gaussian mixture pdf [18] respectively. This approximation reduces complexities in the track management. With the track management including track component pruning and merging [26,27,28,29,30], FIsJITS removes majority of unwanted fJITS and bJITS components. FIsJITS employs track management technique [25] which compares component measurement histories computed in last four scans to merge the identical fJITS and bJITS components respectively. For example, an identical component measurement is repeating in the last four scans as depicted by blue-circle in the ellipsoid fJITS track validation gates from scan *K*th to *K*th + 3 as shown in Figure 4. Therefore, the fJITS track merges these identical components in scan *K*th + 3. Figure 4 also illustrates the component propagation and formation of a new component with a feasible selected measurement outcome in each forward-time scan. FIsJITS applies a same pruning threshold as employed in [25] to remove the fJITS and bJITS components which have a low component existence probability. 

**Figure 5:**
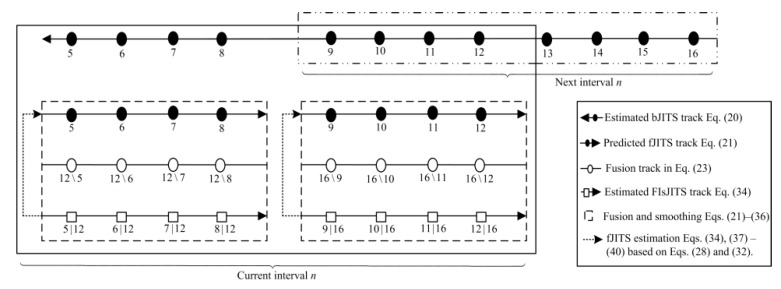
Overlapped smoothing intervals.

Figure 5 shows overlapped smoothing intervals where each fixed-measurement-interval has a length of *n* and consists of *N–B*th + 1 scans. The length of an interval can be chosen depending on various situations. However, higher the length *n*, better would be the estimation which reflects in the RMSE reduction. Let the current measurement-interval includes *b* = 5, 6, …, 12. First smooth the half of an interval *n*/2 depicted by dashed-line and compute the fJITS estimates depicted by dotted-arrow-line in Figure 5. This half of smoothing interval should be discarded so that the next subsequent measurement-interval can be overlap in the remaining half of an interval. This technique limits the time-delay and results in the maximum smoothing. For example, the next measurement-interval includes *b* = 9, 10, …, 16, where bJITS takes a new start from scan *b* = 16 to *b* = 9. However, fJITS track recursion starts from scan *K*th = 9 (when *B*th = 9) using the predicted forward track components from *K*th − 1 (e.g., *K*th = 8). Similarly, smoothing is obtained from *k* = 9, …, 12 conditioning on the last scan measurements in ***Y****_N_* = ***Y***_16_. In this example, there are eight scans in each interval where four eldest scans are discarded after smoothing and four new scans are appended before smoothing the next subsequent interval. 

**Figure 6:**
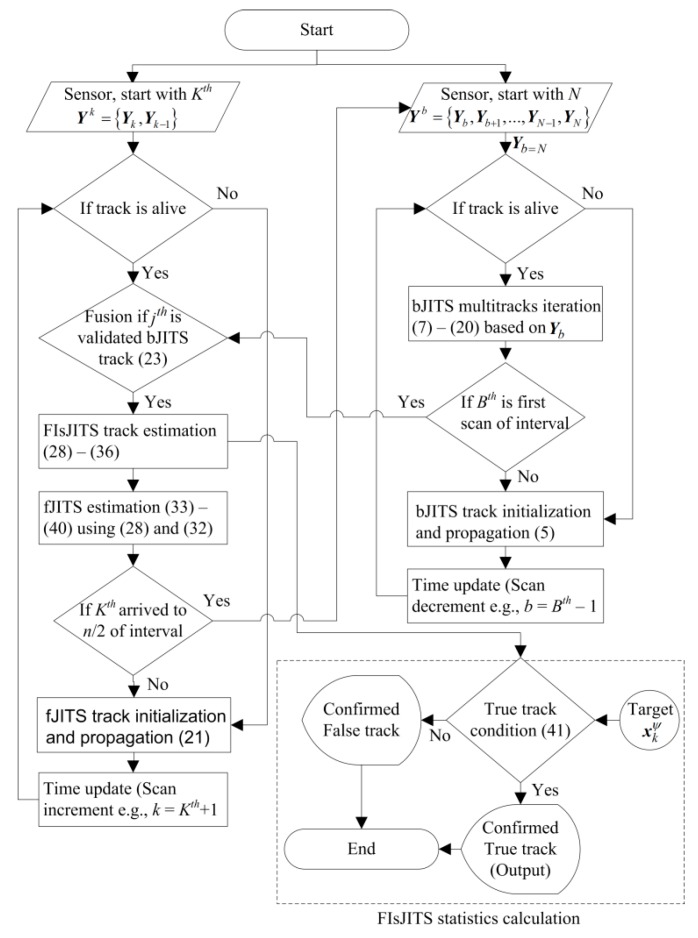
Flow-chart of FIsJITS.

Figure 6 illustrates flow-chart of the two-way tracking and smoothing algorithm. The algorithm starts with backward filtering, giving the measurements ***Y****^b^* to the bJITS. The bJITS use the measurement set of ***Y****_b=N_* and ***Y****_b=N_*_−1_ (two-point initialization) to initialize the tracks required for backward estimation in *B*th scan using (7–20). Therefore, there is no track alive in *b* = *N* and *b* = *N* − 1 and the track recursion starts from *B*th + 1 and continues in reverse direction until it arrive at the first scan of an interval. If a fJITS track is alive at *K*th scan, it generates a predicted FIsJITS track associated to validated bJITS track. Consequently, a FIsJITS and a fJITS track estimates are obtained using (28) and (32) in the *K*th scan. Repeat (21) to (40) recursively to obtain smoothing and forward estimates in each scan until *n*/2 length of an interval is arrived. 

For FTD, FIsJITS uses track quality measure referred to smoothing target existence probability which confirm the track if it exceeds the predetermined confirmation threshold, otherwise the track is terminated. Similarly, the updated target existence probability is employed in bJITS and fJITS for track quality measure in backward and forward tracks, respectively. Each confirmed track remains confirmed until termination. The estimates of the confirmed FIsJITS track must be validated through the target states to select only confirmed true track in (41). Otherwise, the confirmed false track not satisfying Equation (41) is terminated.

(41) $$\sigma_{k|N}^{T}P_{o}^{-1}\sigma_{k|N}\leq \gamma$$

where $\sigma_{k|N}=x_{k}^{\psi }-{\hat{X}}_{k|N}^{\psi }$ expresses the difference between the *ψ*th target state expressed in Equation (1) and the *ψ*th smoothed state estimate obtained in Equation (35), $P_{o}$ represents initial covariance matrix of the target noise measurement which was used for track (forward and backward) initialization and γ is determined from the false-alarm probability of chi-square distribution [2] and [19]. Note that in each scan, new fJITS multi-tracks may be initialized using ***Y****^k^*. These new fJITS track are concatenated with the existing fJITS tracks and the iteration of this algorithm continues in each scan as illustrated in the flow-chart in Figure 6.

## 4. Numerical Analysis Using Simulations

**Figure 7:**
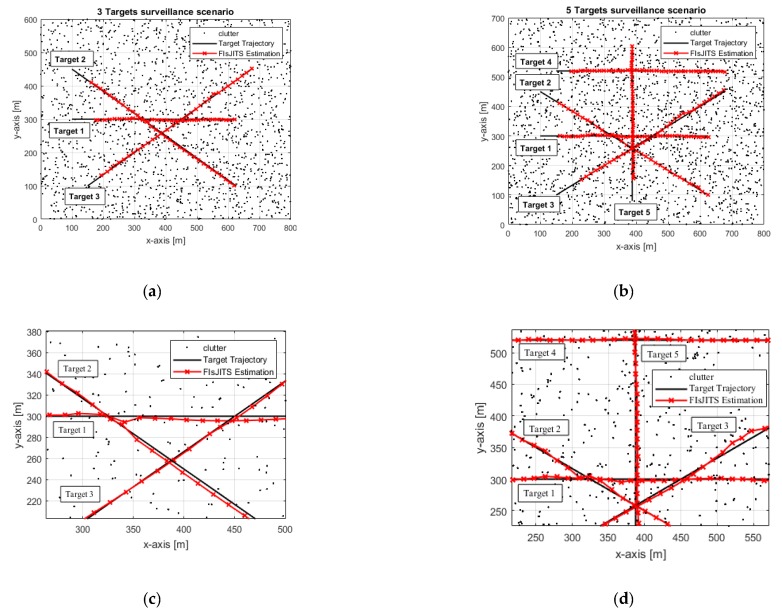
Surveillance scenarios in Clutter (**a**) Three cross-over targets (**b**) Five cross-over targets (**c**) Three cross-over targets (Zoom-in) (**d**) Five cross-over targets (Zoom-in).

Multiple cross-over targets in the two-dimensional surveillance region are demonstrated for numerical simulation as shown in Figure 7. The FIsJITS is analysed for three cross-over targets in the (800 m, 600 m) area and five cross-over targets in the (800 m, 700 m) area. These targets are moving in the heavy clutter environment which is associated to the clutter measurement density *ρ_k_*_,*i*_ = 1 × 10^−4^ m^2^. Table 2 lists an average number of fJITS and bJITS tracks, number of sensor measurements and number of measurements associated to tracks in a validation gate. For example, in five cross-targets scenario, there are 36 (average) measurements received per scan. Two-point initialization method stated that a pair of measurements in consecutive scans satisfying the target maximum velocity limit (i.e., 25 m/s) initializes a track. This generates 16 forward tracks and 27 backward tracks in average, respectively. However, there is/are zero or 3 measurements (average outcome, *i* > 0) in each track validation gate which may be originated from a target (true track) or a clutter (false track). The *ψ*th target position measurement is detected with *P_d_* = 0.9 and is correlated with its noise measurement having known covariance ***R****_k_ =* 25***I***_2_ m^2^ where ***I***_2_ is 2×2 identity matrix. In this environment, the execution time of the algorithms depends on various factors including the target detection probabilities, number of targets, target uncertain motions, non-uniform clutter measurement density and number of initialized tracks in the surveillance region. The average execution time per run of the algorithms is listed in Table 3. JIPDAS consumes more computational time, because of increasing number of measurement-to-track allocations and their a-posteriori probabilities calculation. Compared to this, FIsJITS removes majority of components (in fJITS/bJITS tracks) which reflects in reduction of computational complexity. Track component is removed if updated component existence probability dips below the predetermined pruning threshold. For a fair comparison of the algorithms, similar pruning threshold is applied to FIsJITS, JITS and ITS-S. 

The tracking estimation with FIsJITS is compared with that of the existing smoothing/non-smoothing MTT algorithms based on JIPDAS, JIPDA, JITS and STT algorithm based on ITS-S to verify FTD performance of FIsJITS. The numerical simulation is tested for 500 runs. There are 36 scans with sampling interval of *T* = 1 s per scan in a run. Table 4 shows an initial position measurement of the targets. Each target appears at a different time and moves with an initial velocity of 15 m/s in such a way that it cross-over other targets at different times and angles as shown in Figure 7. For example: targets 1 and 2 in Figure 7a appear at 1 sand target 3 appears at 4 where they cross-over in scans 16, 20 and 24 (with respect to Target 1 appearance). In Figure 7b, another two targets (target 4 appears at 4 s and target 5 appears at 1 s) enter the surveillance region where target 5 cross-over Targets 2 and 3 in scan 13, Target 1 in scan 16 and Target 4 in scan 30. The two surveillance scenarios depicted in Figure 7a–d verify the effectiveness of the smoothed estimations obtained from the FIsJITS algorithm for tracking multiple cross-over targets in heavy clutter.

**Figure 8:**
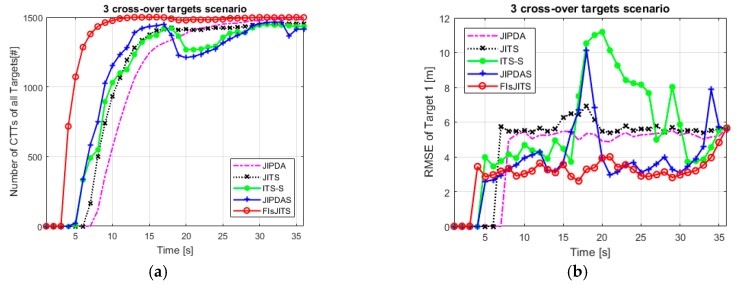
Three cross-over targets (**a**) Number of CTTs (**b**) RMSE of target 1.

The target state transition probability [17] is predefined by α=0.98 which is used for track initialization and propagation in the forward and backward paths. For a tangible FTD comparison using (41), use the same interval length for FIsJITS, JIPDAS and ITS-S algorithms and regulate the confirmation limit of smoothing/non-smoothing algorithms until a similar number of confirmed false tracks (≈27) are obtained. Figure 8 shows the number of confirmed true tracks (CTTs) of three cross-over targets which emphasizes the improved FTD performance of FIsJITS versus existing algorithms. The result also illustrates that JIPDA, JIPDAS, JITS and ITS-S algorithms confirm the track quite late as compare to that of FIsJITS in such multiple cross-over targets scenario. When the targets are cross-over other in scans 16, 20 and 24, CTTs generated by ITS-S and JIPDAS lost almost 30% of the number of CTTs. Due to joint data association capability in JITS track, JITS is quite effective in the cross-over scans as compare to ITS-S and JIPDAS as depicted in Figure 8a. Introducing the proposed novel idea in the JITS algorithm results in optimum tracking performance in FIsJITS as depicted from scan 10 to end scan in Figure 8a. 

Figure 8b shows the root-mean square position estimation errors (RMSEs) statistics of the Target 1. The insufficient FTD performance of ITS-S and JIPDAS in Figure 8a results in the higher RMSEs as depicted at the cross-over scans. In addition, due to incapability of joint data association in ITS-S, CTTs of ITS-S produce larger estimation errors which risen even over the non-smoothing algorithms depicted from scans 17 to 30. Generally, without smoothing application the tracking algorithm like JIPDA and JITS produce higher estimation errors. In the end scan (*N* = 36), the CTT estimates of all algorithms are calculated based on ***Y****_N_* which tend to taper-off their estimates near the end scan. Thus, the RMSE of the algorithms converged at end scan. Compared to the existing algorithms, the FTD performance of FIsJITS depicted in Figure 8a reduces RMSE error for three cross-over targets scenario as shown in Figure 8b.

**Figure 9:**
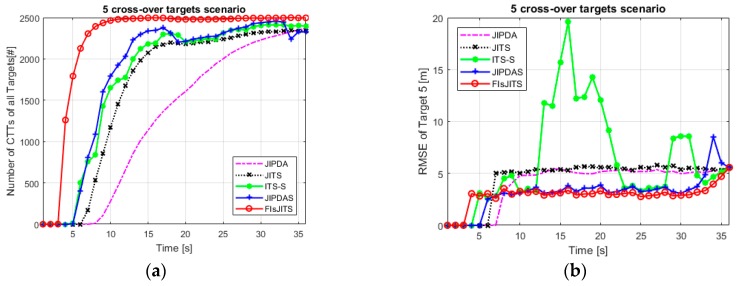
Five cross-over targets (**a**) Number of CTTs (**b**) RMSE of target 5.

Similarly, five cross-over targets in the (800 m, 700 m) surveillance region in Figure 7b are numerically simulated. All other initial parameters like detection probability (*P_d_*), clutter measurement density and an initial position of target 1, 2 and 3 are same. Adding more targets in the surveillance region consume more computational complexities as listed in Table 2 and Table 3. Figure 9a compares tracking performance and FTD capability of five cross-over targets. Compared to the JIPDA and JITS algorithms, the smoothing algorithms performed better. However, JIPDAS and ITS-S are often slow in confirming true tracks in the presence of multi-targets joint measurements and hence missing the targets at regular intervals especially at the cross-over scans. FIsJITS is still provides optimum tracking performance depicted from scan 10 to end scan which reinforces the application of smoothing data association (based on FIsJITS) on JITS.

The RMSE trend of target 5 depicted in Figure 9b is almost same as that of target 1 in Figure 8b except the RMSE statistics of ITS-S and JIPDAS at the cross-over scans. It is noted that in all RMSE cases, the large RMSE of the ITS-S algorithm is obvious especially when the measurement of one target is associated to other targets (at cross-over scans). Moreover, the MTT algorithms produce much larger number of initialized false tracks than that of generated from the STT algorithms. However, in these multiple cross-over targets environment, FIsJITS reduces the estimation error of the targets quite smartly. 

## 5. Conclusions

FIsJITS is the extension of ITS-S in the MTT environment developed for tracking multiple cross-over targets in clutter. The algorithm provides a formula for calculating bJITS joint data association probabilities which was not addressed in the sJITS. A novel approach is utilized where the bJITS *ψ*th target existence probability is used for calculating the predicted smoothing *ψ*th target existence probability. The likelihood ratio of forward and smoothing tracks generated by ITS-S is also modified in FIsJITS for an effective smoothing. The smoothing multi-target data association probabilities are employed for computing both FIsJITS smoothing state and fJITS state estimates. The numerical assessments are analysed using simulation to show almost 100% tracking with FIsJITS for tracking multiple cross-over targets.

## Figures and Tables

**Table 1:** Measurement-to-track assignments and their a-posteriori probabilities in cluster one [2].

PJE (*i* = 1, 2, …)	T1	T2	T3	A-Posteriori Probability
*p*(*ε*_1_|***Y**_b_*)	0	0	0	Equation (14)
*p*(*ε*_2_|***Y**_b_*)	M1	0	0	Equations (14) and (16)
*p*(*ε*_3_|***Y**_b_*)	M2	0	0	Equations (14) and (16)
⋮	⋮	⋮	⋮	⋮
*p*(*ε*_21_|***Y**_b_*)	M2	M3	M4	Equation (16)

**Table 2:** FIsJITS parameter’s specification in number (#).

Scenario	# of fJITS Tracks	# of bJITS Tracks	# of Sensor Measurements	# of Measurements in a Tracking Gate
Three targets	216,000 (≈12 per scan)	378,000 (≈21 per scan)	≈28 per scan	≈2 per forward track in each scan
Five targets	288,000 (≈16 per scan)	486,000 (≈27 per scan)	≈36 per scan	≈3 per forward track in each scan

**Table 3:** Algorithm’s execution time per run (sec).

Scenario	FIsJITS	ITS-S	JITS	JIPDAS	JIPDA
Three targets	3.0	5.5	3.4	6.4	2.5
Five targets	7.6	8.3	6.6	9.5	3.5

**Table 4:** Target’s initial position.

Target #	Initial Position (m)
1	[100; 300; 15; 0]
2	[100; 450; 15; −10]
3	[150; 100; 15; 10]
4	[150; 520; 15; 0]
5	[387.5; 80; 0; 15]

## Nomenclatures

*k* = 1, 2, …, *K*th	Scan index in forward-path tracks.
*b* = 1, 2, …, *B*th	Scan index in backward-path tracks.
*N*	Index of last scan in the smoothing interval which has length of *n*.
*N*\*k*	Scan index where *k* is removed from interval when fusing forward and backward predictions.
*k*|*N*	Scan index in which smoothing estimate is obtained conditioning on measurements in *N*.
***Y**_k_*	Sensor measurements in forward-path tracks.
***Y**_b_*	Sensor measurements backward-path tracks.
***Y**_k,i_*/***Y**_b,i_*	*i*th measurement in ***Y****_k_/**Y**_b_* respectively.
***y*** *_b_*_,*i*_	*i*th backward validation measurement selected from ***Y****_b_* in the validation gate created by bJITS track.
${\tilde{y}}_{k,i}$	*i*th smoothing validation measurement selected from ***Y****_k_* in the validation gate created by FIsJITS track.
*ρ_k_*_,*i*_ ≡ *ρ*(***Y****_k_*_,*i*_)	Clutter measurement density of the measurement ***Y****_k_*_,*i*_.
$Y^{k}=\left\{Y_{k},Y_{k-1}\right\}$	Set of two consecutive forward-time scans measurements used for initializing forward tracks.
$Y^{b}=\left\{Y_{b},Y_{b+1},...,Y_{N-1},Y_{N}\right\}$	Set of consecutive scan measurements starting from last scan index *N* to the *B*th scan of an interval and used for initializing backward tracks.
${\hat{\delta }}_{k}^{\psi }\equiv P\left\{\chi_{k}^{\psi }|Y_{k-1}\right\}$	fJITS Estimated target existence probability of the target existence event $\chi_{k}^{\psi }$ (where *ψ* = 1, 2, …, *ψ*th denotes target index) in *K*th scan conditioned on ***Y****_k_*_−1_
${\hat{\delta }}_{b}^{\psi }\equiv P\left\{\chi_{b}^{\psi }|Y_{b+1}\right\}$	bJITS Estimated target existence probability of the target existence event $\chi_{b}^{\psi }$ in *B*th scan conditioned on ***Y****_b_*_+1_.
$\left[{\hat{X}}_{b}^{\psi },{\hat{P}}_{b}^{\psi }\right]$	Denote the mean and covariance of the *ψ*th target state estimate for one of the backward component calculated using ***Y****_b_* in *B*th scan.
$\left[{\bar{X}}_{b+1}^{\psi },{\bar{P}}_{b+1}^{\psi }\right]$	Denote the mean and covariance of the *ψ*th target state prediction for one of the backward component calculated using ***Y****_b_*_+1_ in *B*th + 1 scan.
$\left[{\hat{X}}_{k}^{\psi },{\hat{P}}_{k}^{\psi }\right]$	Denote the mean and covariance of the *ψ*th target state estimate for one of the forward component calculated using ***Y****_k_* in *K*th scan.
$\left[{\bar{X}}_{k-1}^{\psi },{\bar{P}}_{k-1}^{\psi }\right]$	Denote the mean and covariance of the *ψ*th target state prediction for one of the forward component calculated using ***Y****_k_*_−1_ in *K*th − 1 scan.
